# Measuring institutional community engagement: Adding value to academic health systems

**DOI:** 10.1017/cts.2019.373

**Published:** 2019-06-17

**Authors:** Syed M. Ahmed, Sharon Neu Young, Mia C. DeFino, Joseph E. Kerschner

**Affiliations:** 1 Office of the Senior Associate Dean and Associate Provost for Community Engagement, Medical College of Wisconsin, Milwaukee, WI, USA; 2 DeFino Consulting, LLC, Chicago, IL, USA; 3 Office of the Dean and Provost, School of Medicine, Medical College of Wisconsin, Milwaukee, WI, USA

**Keywords:** Community engagement, community-engaged research, academic health systems, measuring institutional community engagement, community engagement dashboard

## Abstract

Beyond medical schools’ historical focus on pillar missions including clinical care, education, and research, several medical schools now include community engagement (CE) as a mission. However, most academic health systems (AHSs) lack the tools to provide metrics, evaluation, and standardization for quantifying progress and contributions of the CE mission. Several nationwide initiatives, such as that driven by the Institute of Medicine recommending advances in CE metrics at institutions receiving Clinical and Translational Science Awards, have encouraged the research and development of systematic metrics for CE, but more progress is needed. The CE components practical model provides a foundation for analyzing and evaluating different types of CE activities at AHSs through five components: research, education, community outreach and community service, policy and advocacy, and clinical care. At the Medical College of Wisconsin (MCW), an annual survey administered to faculty and staff assessed the types and number of CE activities from the prior year. Survey results were combined to create a CE report for departments across the institution and inform MCW leadership. Insights gathered from the survey have contributed to next steps in CE tracking and evaluation, including the development of a CE dashboard to track CE activities in real time. The dashboard provides resources for how individuals can advance the CE mission through their work and guide CE at the institutional level.

For many years, community engagement (CE) or collaborations between institutions of higher education and their larger communities (local, regional, state, national, and global) for the mutually beneficial exchange of knowledge and resources in a context of partnership and reciprocity have been included in the missions of several land-grant universities [1,2]. However to date, CE is only included as a distinct mission in a few [3] medical schools. As CE is acknowledged as an important adjunct to improve the health of the nation through partnerships to reduce health disparities in communities, there is increased importance in including CE as a distinct School of Medicine (SOM) mission [4–9].

In relation to academic health systems (AHSs), most academic institutions have three missions: clinical care, education, and research [10]. CE as a mission touches all other missions[10,11]. CE in research (CEnR) is a research paradigm that involves communities as partners rather than subjects [1,10]. Most institutions may not really connect CEnR with a research mission. CEnR is a research process like any other research (e.g., basic, clinical research) and should be treated in a similar way [1]. In addition to this assumption, the need for translational science, which goes from T1 to T5, requires involvement of larger stakeholders that include communities, and CE is a recognized process of making community inclusion happen [10,12–14]. Integrating CE with the AHSs missions of clinical care, research, and education is essential to realize the benefits of CE and its ability to improve health [1,10,12,13].

In translational science research covering phases T1–T5, the critical aspect is moving research bi-directionally from bench to bedside to curbside [13,15]. To get the community involved (curbside), there is a demand that communities become partners in all stages of research [10,12,13]. CEnR lends itself to make this process move in an effective manner where collaboration and engagement of stakeholders are fundamentally built on the principles of CE [1,12,13]. For translational research to succeed, it needs to utilize the skills of CE practitioners in involving relevant stakeholders [1,10,12,13].

CE moves beyond community outreach and service, which are traditional concepts of AHSs civic responsibilities primarily focused on volunteer activities that are either related (outreach) or unrelated (service) to professional roles [10,11]. Community outreach and community service are primarily unidirectional on a spectrum of CE activities that extend to and are based on principles of bi-directionality between community-academic partners [10,11]. CEnR is a critical component of CE’s larger platform. It is time for AHSs to go beyond community outreach and community service; although valuable, these activities do not necessarily bring community voices into programming or developing research that impacts communities [10,11]. The confluence of the art and science of CE is actualized using CEnR processes [1]. If AHSs need to advance science at the same time as effectively working with communities, then it is important to follow the principles of CE [1,10,12].

In addition to the need to integrate CE activities, CE stakeholders need to understand how CE affects academic institutions and the value of CE to the institution and community [3,16–18]. The imperative for institutional metrics and valuation is not only for institutional interests, but also emerging requirements for CE valuation from external stakeholders (e.g., grant funding agencies and translational science partners). Within the United States, measuring CE activity and effects is a national directive from the NIH, Clinical and Translational Science Awards (CTSAs), Patient Centered Outcomes Research Institute, and the Carnegie Foundation [19–21]. Paired with this directive of measuring CE activities, for example, previous Carnegie Foundation applications have required evidence of infrastructural support for CE programs and activities, including documented policies, financial investment, and marketing and promotion of CE programs and activities.

Medical schools have developed systems for tracking the contributions of clinical care, education, and research missions primarily, as well as for tracking faculty contributions to evaluate promotion and tenure status [4,22–25]. A mission may or may not generate a positive financial margin for the medical school or AHS, but each medical school mission positively contributes to improving society [26], and its value should be considered on multiple levels. For CE, there are measurements that have been described on a project basis and the factors that contribute to partnership success (context, trust, reciprocity, power dynamics, bi-directional communication, others) have been established [5,27–30]. Although some institutions have taken the initiative to document and catalog the extent of their CE activities [16,18,31], it is uncommon among AHSs or medical schools to have a deep understanding of the types and number of CE activities that occur in their institution [4,16,25,32].

Most institutional processes that have been developed to track, measure, and value CE activities lack the robust, comprehensive, and detailed data comparable to other AHS missions, which limits the institution’s ability to provide accurate assessments to consider the contributions of CE activities to the institution. Therefore, it is critical for AHSs to take next steps in developing systems and processes to integrate CE tracking and metrics similar to other missions. CE activities related to research, education, funding, publications, and community change also need to be accounted for during the promotion and tenure process for faculty that focus primarily on CEnR and community-academic partnership projects for health. Developing new systems and processes for the institution can create administrative burden and requires that staff and faculty have a vested interest in the outcomes. This article describes one approach to measuring institutional CE and provides recommendations for future metrics and tracking CE.

The purpose of this paper is to describe the Medical College of Wisconsin’s (MCW’s) approach to measuring institutional CE as part of MCW’s mission; limitations of and challenges with an CE annual survey; and creating a dashboard for enhancing tracking, measuring, and valuing CE.

To advance CE as a mission in the MCW SOM and to further define institutional CE, a model, the CE components practical model, has been developed.

## The CE Components Practical Model

**Fig. 1.**
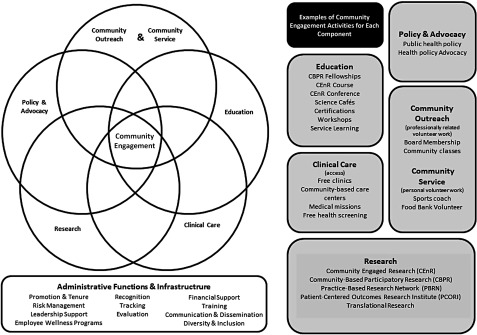
The community engagement components practical model [11].

The CE components practical model (Fig. 1) provides a foundation for analyzing different types of CE activities through its five components: research, education, community outreach and community service, policy and advocacy, and clinical care [11]. Overall, the model outlines different criteria for which activities are related to each component and proposes that not all CE activities fall under one component, but that there is the potential for activities to have overlapping components. The model includes institutional infrastructure and administrative functions that contribute to supporting and sustaining CE activities (such as promotion and tenure, tracking, and evaluation).

## Measuring the Baseline of Institutional CE

At MCW, the Office of the Senior Associate Dean (SAD) for Community Engagement (OCE) led the development of survey questions to assess the baseline activity of institutional CE. An important part of establishing the institutional imperative and importance of the CE mission at MCW included the creation of an Office and SAD role in the medical school leadership team which was on par with the other recognized missions of research, education, and clinical care.

### Survey Questions

The survey was developed based on: (1) background and expertise of the researchers associated with the OCE, (2) the Carnegie Classification Framework and literature, (3) specific definitions and references on CE activities from the literature, (5) defining questions around the CE mission, (6) strategic institutional interests, (7) feedback from the general community at MCW and collaborating institutions, and (8) evolution of the questions from the previous survey administration (i.e., feedback changed some of the questions for the next survey). Overall, the authors’ collective experience in developing and implementing CE programs helped develop the survey to track CE activities that faculty and staff do in communities. As tracking institutional CE is in its nascent state, the survey tool is a first step in attempting to quantify and track CE activities through the creation of administrative data of what is done in the real world.

### Survey Administration

There was a total of 5 questions in the survey administered to faculty (2014) and to staff (2015) and 10 questions for the survey in the years that followed (2016 – faculty and 2017 – staff). Administration of the survey alternated years between faculty and staff. The survey was open for individuals to submit responses for ≅30 days regarding CE activities from the past 12 months. The online survey was distributed via a unique survey link sent directly to each individual’s MCW e-mail address. In 2016/2017, those individuals who had submitted responses in 2014/2015 had the opportunity to review data entered from the previous submission, approve the data that were still relevant, or delete data that were no longer relevant. After survey participants reviewed previous data, they entered any new data for the year. Data were collected between 2014 and 2017 and were analyzed in 2018. This study was approved by the IRB at the MCW under protocol PRO00027876.

### Survey Results

In 2014, there were 656/1584 (41.4%) faculty responses and in 2016, 896/1688 (53.1%) responses. The total number of CE activities reported each year increased by 3.2-fold (478 vs 1507). In 2014, 282 faculty reported 478 CE activities, suggesting that ≅1.7 activities were reported per faculty (Table 1), whereas in 2016, 381 faculty reported 1507 CE activities, suggesting that ≅4 activities were reported per faculty, a 235% increase. The faculty reporting CE activities were from 23/26 departments^a^ in 2014 and 33/35 departments in 2016, representing involvement from across the institution.

**Table 1.** Number of activities reported by faculty and staff

Type of Community Engagement (CE) Activity	Faculty		Staff	
	2014 (*n* = 282)	2016 (*n* = 381)	2015 (*n* = 321)	2017 (*n* = 421)
Outreach and Service*	247	776	451	664
Education	94	157	93	171
Research	51	130	44	42
Clinical Care	NA	67	NA	51
Policy and Advocacy	NA	101	NA	69
Publications and Presentations	35	110	34	38
Awards and Events	51	166	64	301
Total Activities Reported	478	1507	686	1336

* In 2016/2017, service activities were asked about in a separate question from outreach activities.

NA, Not applicable; in 2014/2015, these activities were not asked about.

For staff, the response rate decreased between each year (2015: 56% [1690/3026] vs 2017: 34% [1318/3894]) and the total number of CE activities reported increased by 1.9-fold (686 vs 1330). In 2015, 321 staff reported 686 CE activities, suggesting that ≅2 activities were reported per staff member. In 2017, 421 staff reported 1330 CE activities, suggesting that ≅3.2 activities were reported per staff member, a 160% increase. The staff reporting CE activities were from 51/54 departments in 2015 and 53/56 departments in 2017, representing involvement from across the institution.

### Types of CE Activities Reported

The most reported activities were in the outreach and community service component, followed by education and awards and events for faculty and staff; increases were observed each year. Table 1 shows the number of specific activities reported each year. For activities related to policy and advocacy, clinical care, and service, there were no comparison data from the 2014/2015 surveys as these questions were added in the 2016/2017 surveys.

## Summary

Although the survey tool is limited in the clarity of the construct being measured (i.e., CE as a survey construct has not been thoroughly defined), it is continually being improved, and the data gathered thus far have provided remarkable, unprecedented insights to both the expansiveness and nature of CE activities throughout the institution. A broad distribution of CE activities was reported by faculty and staff throughout MCW. This is contrary to the expectation of the OCE that the results would show CE activities concentrated within specific departments and programs already known to practice CE, and which strongly identify their work as being related to CE.

Although an increase in activities may show evidence of a positive, upward trend in CE and its integration in the MCW community, additional factors affecting the survey population and tool should be considered in interpreting the results. One notable limitation: there are gaps in administration periods, respondents report on CE activities within the last 12-month period, but the survey is issued to respondents only every 2 years. As a result, the survey may not capture CE activities that occur during the gap year between reporting periods. Also, survey results show marked increases primarily in outreach and service types of CE activities. Multiple factors could contribute to this increase, including: (1) added language to a survey question for years 2016/2017, to explicitly include community service activities, which was absent in 2014/2015 surveys; (2) increase in survey participation; (3) increased awareness of the survey; (4) increase in education and understanding of CE and types of activities; and (5) the addition of two new regional campuses and the School of Pharmacy, increasing the size of the MCW population surveyed.

In addition to capturing CE activities, the survey included feedback questions that were modified each year to gather input relevant to specific MCW CE initiatives and strategic plans, including the CE mission and survey. This sampling of responses to feedback questions speaks to the value of having CE activities visibly tracked and measured: Staff: (1) “I endorse MCW’s efforts to quantify the levels of community engagement that it’s faculty, staff and students engage in. It helps MCW send a positive message to the public and it recognizes and credits MCW members”; (2) “I am very happy to see Community Engagement becoming a bigger priority for MCW, and I am eager for additional opportunities to serve both MCW and our communities. Thank you for moving this mission forward!”; (3) “Excellent survey, it is good to show our community support”; and (4) “Thanks. Hope that more people know the efforts of the MCW involvement of community engagement.”

The survey results were shared with institutional leadership and department chairs to convey where CE is the most prevalent in the institution. In addition, the institutional leadership and department chairs were provided with a supporting document that included guidance in how to interpret the results, and how they may use the survey results to support their departments’ CE staff and mission goals.

## Challenges

Respondents also provided comments that address challenges for the institution related to CE and CE tracking and measurement, including annual survey limitations, perceived lack of career growth, lack of buy-in and prioritization for the mission, and need for infrastructural support that compares with tools used for other missions and priorities: Staff: (1) “This survey tool is much too long for someone as active as I am”; (2) “I feel that I spend so much time at work that I don’t know how I would fit in Community work into my schedule. As this is part of the MCW mission, I would love to see it be made a priority”; (3) “Provide more infrastructure that actually supports research and program implementation”; (4) “This seems like an unimportant strategic focus”; and (5) “Many of my colleagues and I are interested in community engagement. However, many of us also feel that it is under-valued (i.e. when it comes to promotions to associate professor or professor) when compared to research, clinical or teaching efforts. Whether or not this is true, I don’t know (maybe there just needs to be some clarification here?).”

The MCW CE survey administration has highlighted several challenges to fully accounting for institutional CE. First, the results are periodic and only available every other year creating difficulty in assessing more proximate interventions and assessments throughout the year. More timely reports of CE activities could be valuable when department chairs are meeting with faculty to assess CE activities or when institutional leadership needs to consider investment strategies or acknowledge high-performing individuals/departments. Second, individuals within the institution often have difficulty perceiving how their participation or prioritization of CE activities are valued and what effect they have on their opportunities for promotion and recognition, or that they are accountable for supporting growth of this mission in their faculty or staff roles. Third, static timepoints of evaluation fail to show how engagement and involvement are evolving and individuals and departments cannot intervene or change their levels of CE based on prior results. Fourth, failure to monitor the consistency and quality of CE efforts can result in not only poor and inconsistent results, but also damaged relationships and result in a decrease in trust from community partners. Finally, while increasing the frequency of data collection, such as an annual survey, may have the positive effect of raising visibility of CE as a purposeful and valued activity, this frequency of data collection will still be in stark contrast to the robust mechanisms used to track and measure other critical operations within an AHS and the other missions of the institution.

## Future Directions

MCW is taking steps to develop infrastructure that will address challenges identified by the survey. The CE dashboard, which is in development, will be a central online repository for individuals to access tools that provide ongoing support for CE work. While many elements will take extensive work and are long-term goals, the envisioned scope of the CE dashboard tool includes:

1. Tools for real-time entry of CE activities to make results timelier.
  - Leaders can use the dashboard to review each faculty’s CE activity at any time, which makes this tracking of CE activities more flexible and accessible (i.e., the timing is not dependent on when the survey is administered).
  - Individual activities can be directly connected to the professional development system to assess progress toward CE-related goals. This creates better alignment with job responsibilities and goals in supporting the CE mission.
2. Access to software specific to CE work, such as partnership management and networking.
3. Resource links, including:
  - forms, best practices for establishing partnerships with the community [2], related policies, training materials, toolkits, and related templates to facilitate CE work;
  - frequently asked questions; and
  - access to MCW’s CE Learning Repository Resource to assist with sharing CE products across institutions and communities.

By creating a dashboard with these existing tools, CE users will have a resource to incorporate in their daily practice of CE activities, including real-time tracking, to hold them accountable, facilitate their work, and provide access to resources.

We have also identified areas in need of development, which would further enhance institutional CE infrastructure:

1. Assessing the downstream effects of CE activities remains a longstanding challenge for institutions for which there is no existing best practice (i.e., no established guideline for measuring the effects of CE activities). One approach has been to apply a common volunteer rate/hourly wage to the number of hours of community outreach and service, which yields a financial figure for what was donated to the community as time [27]. In the era of health economics and health outcomes research, there may be future models developed to accurately account for the effect and value of the CE research and clinical activities. For example, policy and advocacy activities often have a value-added statement to show how much a policy decision will influence the individuals and community downstream.
2. Scorecard metrics are used at several institutions and could be applied to CE activities. Improving the clarity of CE as a construct will help strengthen the measurement of CE through the scorecard. This is a tool already used in other applications and could benefit AHSs if repurposed more specifically for CE to help individual departments measure the level of CE activity and set goals to advance CE. The scorecard of each department would then be used by institutional leadership to review which departments are involved, making improvements, and engaging their faculty and staff in supporting the CE mission.
3. Return on investment (ROI) does not include CE in current metrics and measures presented to board representatives. Either developing a separate ROI metric for CE or revamping formulas to include CE as part of the performance and financial measures would be a powerful step in affirming the priority of CE as a mission to help leaders understand how to value their efforts in supporting the CE mission and present that value of CE appropriately on par with other missions in the institution.
4. Institutions need to develop strategies and processes for bi-directional communication on CE, both institution-wide and to external stakeholders and community partners. Systematic, accessible feedback mechanisms to garner community input that is in-time and relevant is a critical step in holding institutions accountable and being responsive to community partners. Strategies for communicating CE performance to the entire institution, and not just to CE-focused practitioners, is another step in elevating the CE mission in parity with other AHS missions.

How CE is implemented is unique to every institution, and any portfolio of tools will need to be modified per the requirements, goals, and circumstances of the institution. However, the current landscape for AHSs indicates institutions need to approach CE with greater rigor in order to compete and to excel. Beyond the tools listed within the CE dashboard, institutions can look for further innovations similar to those used for other AHS missions that support, catalyze, and improve CE quality and outcomes, as much for the institution’s benefit as for community partners.

## Footnotes

a The number of departments varies for faculty and staff because of HR classifications and assignments; true values are presented for transparency and to make the statement that the majority of departments and centers responded to the survey each year whether faculty or staff were surveyed.
